# Deciduous vs. Coniferous Biomass from Pruning Waste in Composting: Divergent Pathways of Nutrient Release and Heavy Metal Accumulation

**DOI:** 10.3390/plants15152314

**Published:** 2026-07-28

**Authors:** Elena Magheț, Isidora Radulov, Adina Berbecea, Casiana Mihuț, Alina Lațo, Florin Sala, Daniela Poșta, Alexandra Dida Becherescu, Ionuț Dascălu, Olimpia Alina Iordănescu

**Affiliations:** 1 Doctoral School of Engineering of Plant and Animal Resources, Horticulture, University of Life Sciences “King Mihai I” from Timișoara, 119 Aradului Avenue, 300629 Timișoara, Romania; elena.maghet@usvt.ro; 2 Department of Soil Science, Faculty of Agriculture, University of Life Sciences “King Mihai I” from Timișoara, 119 Aradului Avenue, 300629 Timișoara, Romania; isidora_radulov@usvt.ro (I.R.); casiana_mihut@usvt.ro (C.M.); alina_lato@usvt.ro (A.L.); florin_sala@usvt.ro (F.S.); 3 Department of Horticulture, Faculty of Horticulture, Forestry and Plant Biotechnologies, University of Life Sciences “King Mihai I” from Timișoara, 119 Aradului Avenue, 300629 Timișoara, Romania; danielaposta@usvt.ro (D.P.); alexandrabecherescu@usvt.ro (A.D.B.); ionut.dascalu@usvt.ro (I.D.); olimpiaiordanescu@usvt.ro (O.A.I.)

**Keywords:** deciduous, conifers, pruning waste, compost, pH, macro-and microelements, heavy metal

## Abstract

The sustainable management of urban pruning residues is increasingly important for promoting circular bioeconomy practices and reducing green waste disposal. This study evaluated the composting potential of pruning biomass from 18 urban tree species (11 deciduous and 7 coniferous) and assessed the effects of biomass type and feedstock ratio on compost quality. Plant residues were composted with fresh cow manure using two compost ratios: V1 (700 g manure + 100 g plant biomass) and V2 (700 g manure + 700 g plant biomass). Compost quality was evaluated through pH, macroelement (N, P, K, Ca, Mg) and total microelement (Mn, Cu, and Zn) and heavy metal (Pb, Cd, and Ni) concentrations. The composting ratio was the primary factor controlling compost properties. V1 produced alkaline composts (pH 7.96–9.42) with higher nutrient concentrations, whereas V2 generated composts with pH values closer to neutrality (7.39–9.44). Increasing the proportion of plant biomass reduced macronutrient concentrations, with total N ranging from 2.23% in V1 to 0.72% in V2, and potassium decreasing by 50–80% relative to V1. In contrast, micronutrient concentrations increased in V2, reaching 1142.7 mg·kg^−1^ Mn and 481.1 mg·kg^−1^ Zn. Total heavy metal concentrations also increased with greater biomass incorporation, although values generally remained low; the highest Ni concentration (15.77 mg·kg^−1^) was recorded in Paulownia tomentosa compost. Species-specific responses were observed for nutrient release and metal accumulation, highlighting differences between deciduous and coniferous feedstocks. The findings demonstrate that compost quality is primarily determined by the manure-to-biomass ratio, while tree species influence nutrient dynamics and trace element accumulation. Composts with higher manure proportions are suitable for nutrient enrichment and acidic soil amelioration, whereas biomass-rich composts provide enhanced micronutrient contents but require monitoring of heavy metal accumulation.

## 1. Introduction

Composting is widely recognised as one of the most sustainable strategies for managing biodegradable waste, transforming organic residues into stable products that enhance soil quality and support sustainable agriculture. In addition to diverting organic waste from landfills, composting recycles nutrients and organic matter, enhances soil fertility, improves soil structure and water-holding capacity, stimulates beneficial microbial communities, and reduces dependence on mineral fertilisers [1,2]. Numerous studies have also demonstrated that applying compost promotes plant growth and health by improving nutrient availability, increasing soil biological activity, and suppressing certain soil-borne pathogens [1,2]. Consequently, composting is a key component of the circular bioeconomy and an environmentally sound alternative for the valorisation of organic wastes.

A considerable proportion of municipal solid waste comprises biodegradable materials, including urban green waste, household and catering food waste, and food-industry residues [3]. These materials constitute an underutilised resource that can be effectively recycled through composting or anaerobic digestion [4]. Accordingly, the European Union has set ambitious targets to reduce the landfilling of biodegradable waste and promote its recovery [5]. However, recent assessments indicate that implementation remains uneven across European countries, with Romania still facing significant challenges in the valorisation of biodegradable waste despite an appropriate legislative framework [6,7].

Among biodegradable wastes, urban pruning residues are abundant yet largely underexploited. In Romania, substantial quantities of leaves, branches, and pruning residues generated during the maintenance of urban green spaces are still treated as waste rather than valuable compost feedstocks [8,9]. Their disposal not only increases pressure on waste management systems but also results in the loss of considerable amounts of organic matter and plant nutrients. Studies by Parzych et al. (2022) and Álvarez-Alonso et al. (2025) highlighted the high agronomic potential of urban green waste and its importance for circular economy strategies [10,11]. However, compost quality is strongly influenced by feedstock composition. Similarly, Sánchez-Monedero et al. (2018) and Zhang et al. (2016) reported that increasing the proportion of lignocellulosic biomass generally reduces macronutrient concentrations due to dilution and temporary microbial immobilisation associated with high C:N ratios [12,13]. Conversely, leaf biomass is an important source of micronutrients but may also contribute to the accumulation of potentially toxic metals, particularly when originating from urban environments where foliage acts as a biological filter for atmospheric pollutants [10,11,14]. These contrasting effects underscore the importance of developing composting strategies that maximise nutrient recovery while minimising contamination risks [15,16,17,18,19].

The chemical composition of pruning residues varies considerably among tree species, particularly between deciduous and coniferous trees. Previous studies have demonstrated that the chemical composition of pruning residues differs markedly between deciduous and coniferous tree species. Prescott et al. (2000), Sæbø et al. (2012), and Boldrin et al. (2009) reported that deciduous biomass generally decomposes more rapidly because of its lower lignin content and higher nutrient concentrations, promoting faster nutrient mineralisation and compost maturation [20,21,22,23]. In contrast, Berg and McClaugherty (2020) showed that coniferous biomass is characterised by higher lignin concentrations, polyphenolic compounds, and more recalcitrant tissues, resulting in slower decomposition rates, greater compost stability, and different nutrient release patterns [21]. Consequently, feedstock composition plays a key role in determining compost maturity, nutrient dynamics, and trace-element behaviour [20,21,22,23]. However, studies conducted by Farrell et al. (2009), Gotowska et al. (2025), and Tognetti et al. (2005) generally treated urban green waste as a single, homogeneous feedstock, without distinguishing among individual tree species or comparing the composting behaviour of deciduous and coniferous biomass [24,25,26]. Therefore, information on species-specific effects on nutrient release and trace-element accumulation during composting remains limited.

Consequently, significant knowledge gaps remain regarding how different urban tree species affect compost quality and how the proportion of pruning residues in the composting mixture affects nutrient release and trace-element accumulation. Understanding these species-specific responses is essential for optimising compost formulations and producing safe, agronomically valuable compost from urban pruning residues.

We hypothesised that (i) compost quality is primarily determined by the proportions of plant biomass and manure in the composting mixture, whereas (ii) the botanical origin of pruning residues (deciduous versus coniferous species) further modulates nutrient dynamics and heavy-metal accumulation due to differences in litter chemistry and decomposition behaviour.

Therefore, this study aimed to determine how the origin of plant residues (deciduous versus coniferous tree species) and the plant residue-to-manure composting ratio affect compost quality, as evaluated by pH, macro- and micronutrient dynamics, and heavy-metal accumulation in composts prepared from pruning residues of 18 urban tree species. Furthermore, the study sought to identify species-specific differences in nutrient release and trace-element accumulation, providing new insights into the sustainable valorisation of urban pruning residues and supporting the development of composting strategies tailored to different types of woody biomass.

## 2. Results

### 2.1. Compost pH

**Figure 1:**
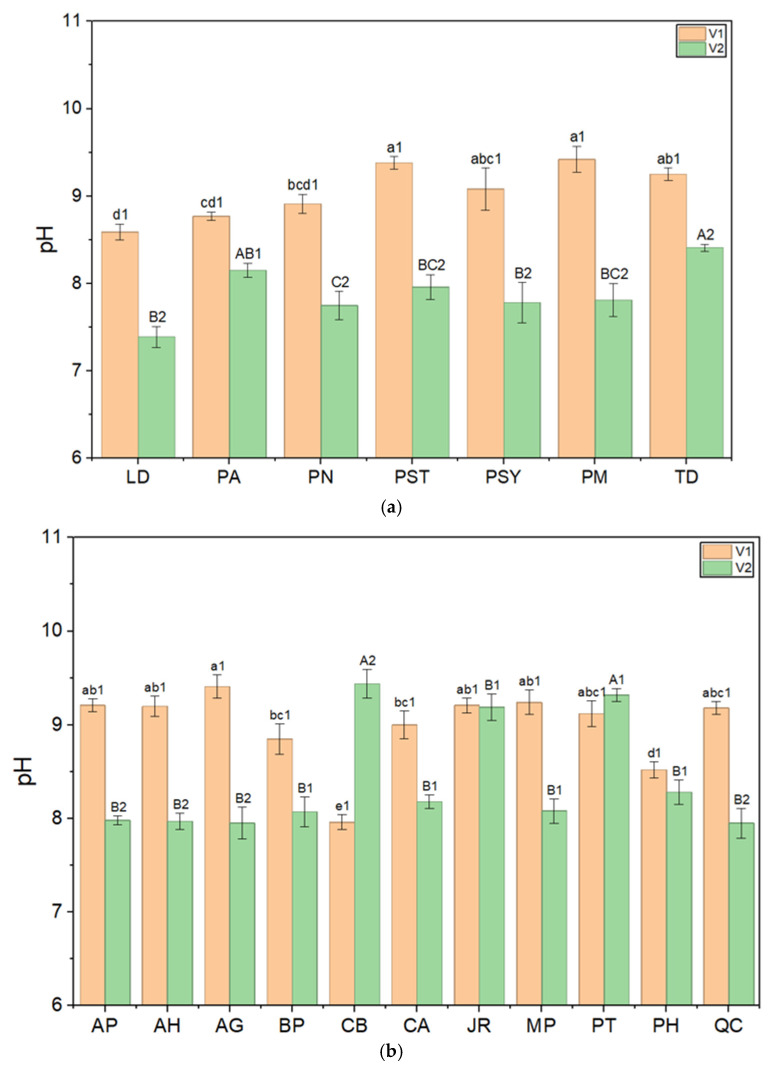
Compost pH ((**a**)—coniferous; (**b**)—deciduous). Means marked with the same letters show no statistically significant differences (*p* > 0.05). Lowercase/uppercase letters are used to denote statistically significant differences between groups within the same variant (V1/V2), whereas numbers indicate statistically significant differences between variants.

Compost pH varied with composting ratio and species, with significant differences between the V1 and V2 variants (Figure 1). In the V1 variant, all composts were alkaline, with pH ranging from 7.96 (*Catalpa bignonioides*) to 9.42 (*Pseudotsuga menziesii*). In the V2 variant, pH ranged from 7.39 to 9.44 and was generally lower, indicating reduced alkalinity with the higher proportion of plant residues. The lowest pH values were recorded for *Larix decidua* (7.39), *Pinus nigra* (7.75), and *Pinus sylvestris* (7.78), whereas the highest values occurred in *Catalpa bignonioides* (9.44) and *Paulownia tomentosa* (9.32).

Most deciduous species exhibited a pH decrease of approximately 1.0–1.4 units in V2 compared with V1, and all coniferous species also showed reduced pH. The greatest decreases were observed in *Pseudotsuga menziesii* (9.42 to 7.81), *Pinus strobus* (9.17 to 7.75), and *Larix decidua* (8.59 to 7.39). In contrast, *Juglans regia* maintained a nearly unchanged pH (9.21 to 9.19), whereas *Paulownia tomentosa* (9.12 to 9.32) and, particularly, *Catalpa bignonioides* (7.96 to 9.44) showed increased pH values in V2.

### 2.2. Macroelements Content of the Compost

**Figure 2:**
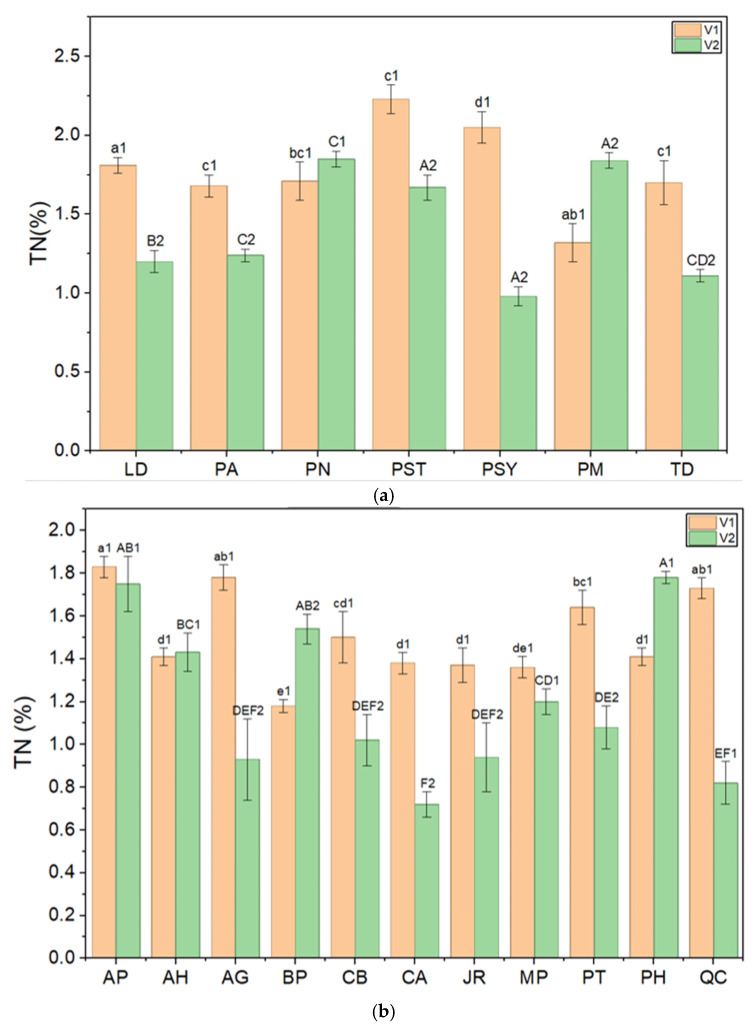
The total nitrogen content (Nt). ((**a**)—coniferous; (**b**)—deciduous). Means marked with the same letters and numbers show no statistically significant differences (*p* > 0.05). Lowercase/uppercase letters are used to denote statistically significant differences between groups within the same variant (V1/V2), whereas numbers indicate statistically significant differences between variants.

Total nitrogen (TN) content varied significantly across composting variants and species (Figure 2). The manure-rich V1 composts had higher TN concentrations than the V2 composts, reflecting the increased proportion of plant residues. In deciduous species, TN ranged from 1.18–1.83% in V1 and 0.72–1.78% in V2, whereas in coniferous species the corresponding ranges were 1.32–2.23% and 0.98–1.85%, respectively. The largest reductions in TN were observed in *Alnus glutinosa* (1.78 → 0.93%), *Catalpa bignonioides* (1.38 → 0.72%), and *Quercus coccinea* (1.73 → 0.82%). In contrast, *Betula pendula*, *Platanus hybrida*, *Pinus nigra*, and *Pseudotsuga menziesii* showed similar or higher TN concentrations in V2 than in V1. The highest TN content was recorded in *Pinus strobus* compost (2.23%) in V1, whereas the lowest value was found in *Catalpa bignonioides* compost (0.72%) in V2, indicating considerable interspecific variability in nitrogen retention during composting.

**Figure 3:**
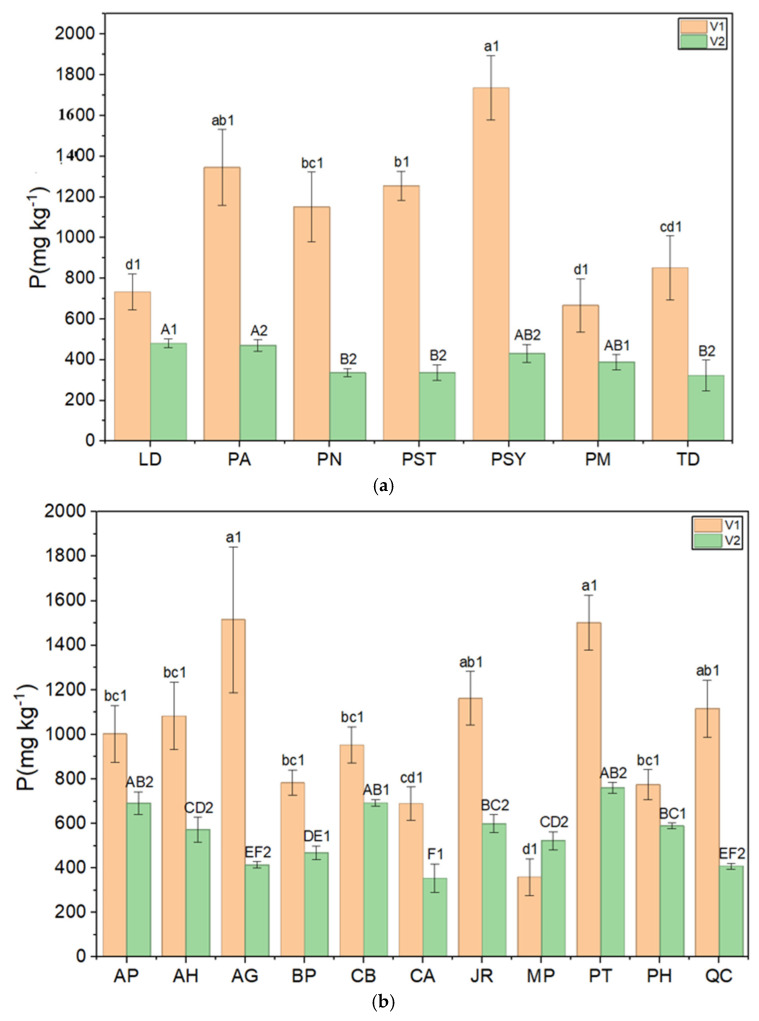
The phosphorus content (P). ((**a**)—coniferous; (**b**)—deciduous). Means marked with the same letters and numbers show no statistically significant differences (*p* > 0.05). Lowercase/uppercase letters are used to denote statistically significant differences between groups within the same variant (V1/V2), whereas numbers indicate statistically significant differences between variants.

Phosphorus (P) content varied significantly across composting variants and species (Figure 3). Overall, V1 composts contained higher P concentrations than V2 composts. In deciduous species, P ranged from 359–1515 mg kg^−1^ in V1 and 354–760 mg kg^−1^ in V2, whereas in coniferous species the corresponding ranges were 667–1736 mg kg^−1^ and 323–482 mg kg^−1^, respectively. The largest reductions in P content were observed in *Alnus glutinosa* (1515 → 414 mg kg^−1^), *Quercus coccinea* (1116 → 409 mg kg^−1^), *Aesculus hippocastanum* (1083 → 573 mg kg^−1^), *Pinus strobus* (1255 → 337 mg kg^−1^), and *Pinus nigra* (1151 → 337 mg kg^−1^). In contrast, *Malus purpurea* was the only species to show an increase in P concentration, from 359 mg kg^−1^ in V1 to 523 mg kg^−1^ in V2.

**Figure 4:**
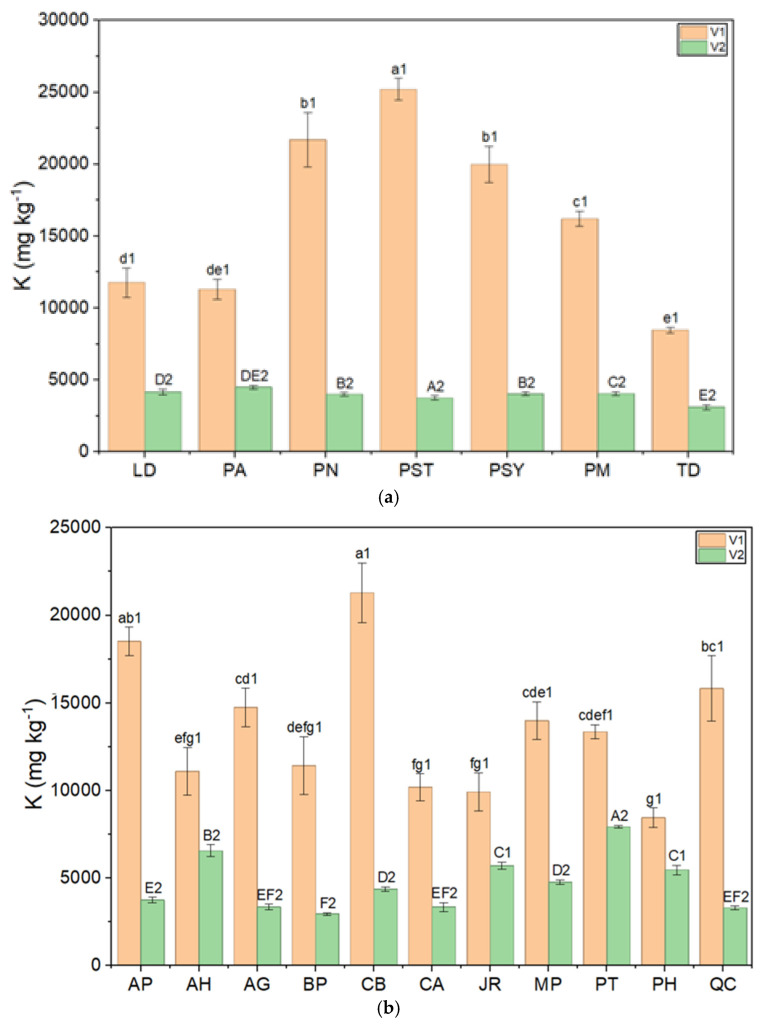
The potassium content (K). ((**a**)—coniferous; (**b**)—deciduous). Means marked with the same letters or numbers show no statistically significant differences (*p* > 0.05). Lowercase/uppercase letters are used to denote statistically significant differences between groups within the same variant (V1/V2), whereas numbers indicate statistically significant differences between variants.

Potassium (K) content differed markedly among composting variants and species (Figure 4). In the V1 composts, K concentrations ranged from 8447–21,289 mg kg^−1^ in deciduous species and 8469–25,196 mg kg^−1^ in conifers, with the highest values recorded for *Catalpa bignonioides* (21,289 mg kg^−1^) and *Pinus sylvestris* (25,196 mg kg^−1^). In the V2 variant, K concentrations declined by approximately 50–80% in most composts, with values generally below 5000 mg kg^−1^. The largest decreases were observed in *Acer platanoides* (18,510 → 3755 mg kg^−1^) and *Pinus strobus* (25,196 → 3748 mg kg^−1^). Although deciduous species exhibited greater variability in K content than conifers, *Paulownia tomentosa* was a notable exception in V2, maintaining a relatively high K concentration (7931 mg kg^−1^).

Calcium (Ca) concentration varied markedly among composting variants and species (Table 1). In the V1 composts, Ca concentrations ranged from 4540 to 17,342 mg kg^−1^ in deciduous species and from 2800 to 20,415 mg kg^−1^ in conifers, with the highest values recorded for *Picea abies* (20,415 mg kg^−1^) and *Acer platanoides* (17,342 mg kg^−1^). Increasing the proportion of plant residues in the V2 composts generally reduced Ca concentrations, with pronounced decreases observed in *Acer platanoides* (17,342 → 6675 mg kg^−1^), *Alnus glutinosa* (14,918 → 5032 mg kg^−1^), and *Picea abies* (20,415 → 6521 mg kg^−1^). In contrast, *Pinus sylvestris* showed an increase in Ca concentration from 2800 to 5757 mg kg^−1^.

Magnesium (Mg) concentrations showed lower interspecific variability than Ca (Table 1). In the V1 composts, Mg ranged from 1046 to 5329 mg kg^−1^, whereas in the V2 composts most species had concentrations between 460 and 500 mg kg^−1^. Overall, both Ca and Mg concentrations were generally higher in the V1 composts than in the V2 composts.

### 2.3. Microelements Content of the Compost

The concentrations of microelements varied across species and composting variants (Table 2). Overall, the V2 composts contained higher concentrations of Mn, Zn, and Cu than the V1 composts, although species-specific responses were observed.

Manganese (Mn) concentrations in the V1 composts ranged from 205.5 to 606.0 mg kg^−1^ and generally increased in V2, reaching 1142.7 mg kg^−1^ in *Pinus strobus* and 691.4 mg kg^−1^ in *Acer platanoides*. By contrast, *Larix decidua* and *Juglans regia* had lower Mn concentrations in V2 than in V1.

Zinc (Zn) concentrations ranged from 49.5 to 133.0 mg kg^−1^ in V1 and increased markedly in most V2 composts, with the highest values recorded for *Juglans regia* (481.1 mg kg^−1^) and *Betula pendula* (332.3 mg kg^−1^). Only *Catalpa bignonioides* showed a slight reduction in Zn concentration in V2.

Copper (Cu) concentrations were lower than those of Mn and Zn, ranging from 11.4 to 27.9 mg kg^−1^ in V1. Similar to the other microelements, Cu generally increased in V2, reaching 90.7 mg kg^−1^ in *Malus purpurea* and 45.7 mg kg^−1^ in *Acer platanoides*, although *Catalpa bignonioides* and *Betula pendula* exhibited comparable or slightly lower values than in V1.

### 2.4. Heavy Metal Content of the Compost

The concentrations of Pb, Cd, and Ni varied among composting variants and species (Table 3). The V2 composts showed higher concentrations of all three metals than the V1 composts. 

Lead (Pb) concentrations were low in all composts but increased consistently in V2. Pb values ranged from 0.89–1.85 mg kg^−1^ in V1 and 2.61–4.59 mg kg^−1^ in V2, with larger increases generally observed in deciduous than in coniferous species. For example, Pb concentration increased from 1.26 to 3.47 mg kg^−1^ in *Acer platanoides* and from 0.89 to 2.61 mg kg^−1^ in *Pseudotsuga menziesii*.

Cadmium (Cd) concentrations remained low, with values below 0.33 mg kg^−1^ in V1 and below 1.0 mg kg^−1^ in V2. Most species exhibited higher Cd concentrations in V2, including *Alnus glutinosa* (0.33 → 0.91 mg kg^−1^) and *Pinus strobus* (0.21 → 0.72 mg kg^−1^), whereas Cd was not detected in *Catalpa bignonioides*, *Juglans regia*, or *Paulownia tomentosa* in either composting variant.

Nickel (Ni) exhibited the greatest interspecific variability. In most species, Ni concentrations in V2 were two- to fourfold higher than in V1, with the highest value recorded for *Paulownia tomentosa* (15.77 mg kg^−1^). In contrast, conifer-derived composts generally maintained low Ni concentrations, with *Picea abies* showing values of 0.30–0.41 mg kg^−1^.

### 2.5. Principal Component Analysis

PCA is applied to reduce dataset complexity and highlight the principal components that explain most of the variability, thereby facilitating interpretation of relationships among variables and the grouping of samples according to their shared characteristics. 

**Figure 5:**
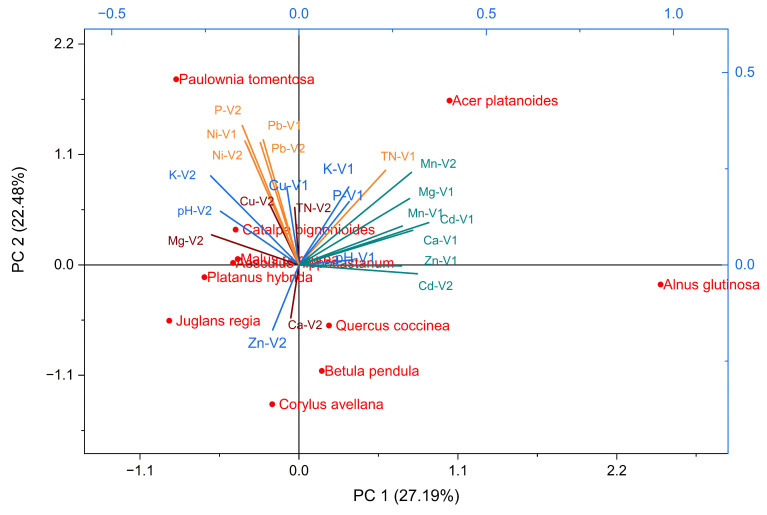
Principal component analysis (PCA) biplot illustrating the relationships between deciduous tree species and the chemical characteristics of the resulting composts under the two composting variants (V1 and V2).

For deciduous residues compost, based on the Eigenvector values, 3 Principal components were identified, which together account for 65.49% of the total variation. PC1 contributes 27.19% of the total variation, PC2 another 22.48%, and PC3 another 16.11% (Figure 5).

For deciduous composts, the first three principal components explained 71.4% of the total variance (Figure 5). PC1 accounted for the largest proportion of the variance and was mainly associated with Cd (V1, V2), Mn (V1, V2), Zn (V1), Mg (V1), and Ca (V1), reflecting a gradient in metal and nutrient concentrations among the composts. Species such as *Alnus glutinosa* and *Acer platanoides* were located on the positive side of PC1 and were associated with higher values of these variables, whereas *Paulownia tomentosa*, *Juglans regia*, and *Platanus hybrida* were positioned on the negative side of the axis.

PC2 was primarily influenced by Pb (V1, V2), Ni (V1, V2), P (V2), and total nitrogen (TN-V1), separating composts according to trace metal and nutrient composition. *Paulownia tomentosa* and *Acer platanoides* were associated with the positive side of PC2, while *Betula pendula*, *Corylus avellana*, and *Quercus coccinea* were located on the negative side. PC3, although not represented in the two-dimensional ordination, was mainly associated with Cu (V2), Mg (V2), Ca (V2), and TN (V2), indicating additional variation related to nutrient composition.

**Figure 6:**
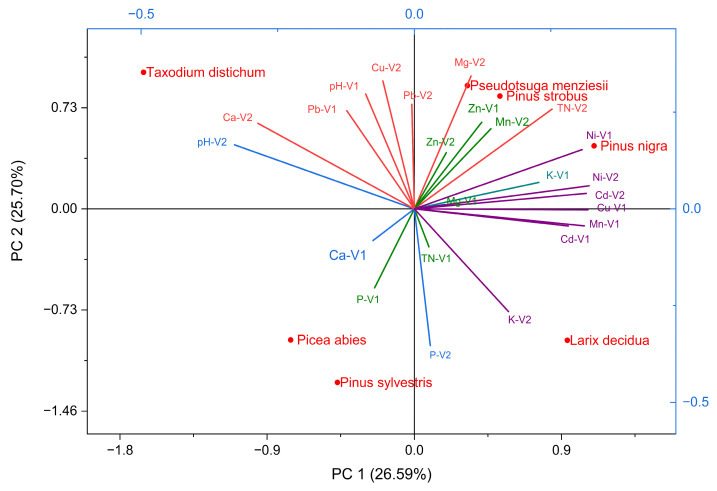
Principal component analysis (PCA) biplot illustrating the relationships between conifers tree species and the physicochemical and elemental characteristics of the resulting composts under the two composting variants (V1 and V2).

For conifer-derived composts, the first three principal components explained 69.5% of the total variance (Figure 6). PC1, PC2, and PC3 accounted for 26.59%, 25.70%, and 17.23% of the total variance, respectively. The ordination separated composts according to differences in nutrient and trace element composition, reflecting species-specific variation in compost chemical characteristics. (Figure 6).

The first principal component (PC1) was primarily associated with Cd (V1, V2), Mn-V1, Ni (V1, V2), Cu-V1, and K-V2, reflecting a gradient dominated by trace-metal accumulation and potassium content. Species positioned on the positive side of PC1, such as *Pinus nigra*, *Pseudotsuga menziesii*, and *Pinus strobus*, were strongly associated with these variables, indicating a greater capacity for metal accumulation. By contrast, *Taxodium distichum*, located on the negative side of PC1, exhibited a distinct profile characterised by lower levels of these elements.

The second principal component (PC2) was defined by Pb (V1, V2), pH-V1, Cu-V2, Ca-V2, Mg-V2, and TN-V2, reflecting a combined gradient in soil chemical properties and nutrient status. Species such as *Pseudotsuga menziesii*, *Pinus strobus*, and *Taxodium distichum*, positioned on the positive side of PC2, were associated with higher values of these variables, suggesting an influence of soil conditions and nutrient availability. Conversely, *Pinus sylvestris*, *Picea abies*, and *Larix decidua*, located on the negative side of PC2, were characterised by lower values or by different nutrient–soil interactions.

Although not directly represented in the two-dimensional ordination, the third principal component (PC3) was characterised by TN-V1, P-V1, K-V1, Mg-V1, Zn (V1, V2), and Mn-V2, capturing additional variability in macronutrients and essential trace elements.

## 3. Discussion

The proportion of manure to plant residues was the primary determinant of the composts’ chemical composition. The V1 mixture, with a higher proportion of cattle manure (plant residue:manure ratio 1:7), consistently produced composts with higher macronutrient concentrations than the V2 mixture (1:1). This response is expected because cattle manure is considerably richer in readily available nutrients than lignocellulosic pruning residues and decomposes more rapidly, promoting nutrient mineralisation during composting [1,27]. In contrast, increasing the proportion of plant residues dilutes the nutrient-rich fraction and increases the input of structural carbon, slowing decomposition and enhancing microbial immobilisation, particularly of nitrogen and phosphorus [21,28]. Similar responses have been reported by Bernal et al. (2009), Awasthi et al. (2020), and Tampio et al. (2016), who demonstrated that manure-rich compost mixtures generally contain higher concentrations of plant-available macronutrients than those produced from woody biomass [1,19,29]. This general pattern underpins the interpretation of the behaviour of the individual nutrients discussed below.

The composting ratio emerged as the main factor controlling compost pH. Increasing the proportion of cattle manure promoted alkaline conditions, whereas greater incorporation of leaf biomass shifted the pH toward neutrality. This response is consistent with the enhanced ammonification and nitrogen mineralisation occurring in manure-rich substrates, which increase ammonia production and alkalinity during composting [1,27]. Conversely, leaf-rich mixtures contain larger amounts of lignin and polyphenolic compounds that decompose more slowly and generate organic acids, resulting in lower pH values [20,28]. The influence of litter chemistry was further demonstrated by the marked species-specific differences. Deciduous species rich in base cations generally maintained higher pH, whereas conifer-derived composts exhibited greater acidification, reflecting the lower buffering capacity and more recalcitrant nature of conifer litter [18,21]. The increase in pH observed for *Catalpa bignonioides* suggests that the chemical composition of certain leaf residues may override the effect of substrate proportion, emphasizing that both compost formulation and feedstock quality determine the final compost reaction.

The manure-to-plant residue ratio was the main factor controlling nitrogen availability in the composts. The higher TN concentrations in the manure-rich V1 mixtures can be attributed to the greater input of readily mineralisable organic nitrogen, whereas increasing the proportion of plant residues in V2 promoted nitrogen immobilisation through higher C:N ratios and the slower decomposition of lignocellulosic materials [1,30]. This mechanism has been widely reported for composts containing woody biomass, where microbial demand for nitrogen during the degradation of recalcitrant carbon reduces the amount of plant-available N [21,30]. Species-specific responses further demonstrate the importance of litter quality. Composts derived from species with lignin- and polyphenol-rich residues tended to retain lower TN, whereas more labile leaf materials maintained or even enhanced nitrogen availability. Similar effects of plant residue chemistry on nitrogen mineralisation have been reported by Hobbie et al. (2012), who identified substrate quality as a key regulator of decomposition and nutrient cycling [31]. The smaller reduction in TN observed for some conifer species suggests that differences in residue composition may also influence nitrogen retention during composting. The present findings agree with those of Álvarez Alonso et al. (2025) [11], confirming that nitrogen dynamics are governed by the combined effects of feedstock composition and species-specific litter characteristics.

Phosphorus availability was strongly influenced by the proportion of manure and plant residues in the compost mixture. The greater P concentrations in the V1 composts reflect the higher phosphorus content and lower mobility of manure-derived P, whereas increasing the proportion of carbon-rich plant residues diluted the nutrient pool and reduced P availability [1,32]. In addition, the higher C:P ratio of the V2 mixtures likely enhanced microbial immobilisation during decomposition, temporarily retaining phosphorus within microbial biomass and limiting its release into the mature compost [19,33]. Similar effects of lignocellulosic plant residues on phosphorus dynamics have been reported by Sanchez-Monedero et al. (2018) and Zhang and Sun (2016), who showed that increasing the proportion of woody biomass decreases nutrient density and delays phosphorus mineralisation [12,13]. Species-specific differences further indicate that the chemical composition of the plant residues influences P release during composting, consistent with the observations of Berg and McClaugherty (2020) and Hobbie et al. (2012), who identified plant residue quality as a major driver of nutrient cycling [21,31]. The increase in P concentration observed for Morus alba suggests that more readily decomposable plant residues may promote phosphorus mineralisation even in mixtures with a higher proportion of plant biomass, as previously reported by Grigatti et al. (2019) [34]. Overall, these findings indicate that both the composition of the composting materials and the chemical characteristics of the plant residues regulate phosphorus availability and should be considered when producing composts for different agricultural applications.

Potassium availability was strongly influenced by the proportion of manure and plant residues in the compost mixture. The higher K concentrations observed in the V1 composts reflect the substantial contribution of manure, which is a readily available source of exchangeable potassium, whereas increasing the proportion of lignocellulosic plant residues diluted the nutrient pool and reduced the overall K concentration [11]. Similar responses have been reported for green-waste composts, where manure-rich mixtures generally contain higher potassium concentrations than composts produced predominantly from woody biomass [35,36,37]. Although the composting ratio was the dominant factor, species-specific differences indicate that the chemical composition of plant residues also influences potassium release during decomposition. The relatively high K concentrations maintained in composts derived from several conifer species suggest that nutrient mobilisation depends not only on decomposition rate but also on the initial potassium content of the plant material, consistent with the findings of Bojarski et al. (2023) and Silva et al. (2024) [38,39]. Likewise, Parzych (2022) reported greater variability in potassium concentrations among deciduous species than among conifers, highlighting the importance of species selection when producing composts with specific nutrient characteristics [10]. Our findings demonstrate that both the composition of the compost mixture and the chemical characteristics of the plant residues determine potassium availability and should be considered when tailoring composts for different agricultural applications [2,29,40,41].

The lower Ca and Mg concentrations in the V2 composts further support the influence of the manure-to-plant residue ratio on the availability of basic cations. Because cattle manure is generally richer in readily available Ca and Mg than woody plant residues, increasing the proportion of plant biomass diluted these nutrients in the compost mixture, consistent with the findings of Domene et al. (2025) [42]. Species-specific differences indicate that the chemical composition of the plant residues also influenced nutrient dynamics. Calcium concentrations varied considerably among species, reflecting differences in the accumulation of structural cations within plant tissues, whereas magnesium appeared to be more strongly affected by the composting ratio because of its greater mobility during decomposition. Similar relationships between plant residue quality, decomposition, and nutrient release have been described by Berg and McClaugherty (2020) and Hobbie et al. (2012) [21,31]. The contrasting responses observed among conifer-derived composts further suggest that differences in tissue chemistry and the degree of lignification regulate the release and retention of these cations during composting [13,20,21]. From a practical perspective, composts enriched with manure may provide a valuable source of Ca and Mg for improving soil fertility and buffering capacity, whereas mixtures containing higher proportions of plant residues may be more suitable where a gradual nutrient supply is desirable [29]. Overall, these findings demonstrate that both the composition of the compost mixture and the chemical characteristics of the plant residues are important factors governing Ca and Mg availability in mature composts [10,11,42].

The higher Mn, Zn, and Cu concentrations observed in V2 indicate that leaf biomass was the primary source of micronutrients in the compost. This finding is consistent with previous studies showing that leaf-rich composts accumulate greater amounts of trace elements because foliage contains higher concentrations of micronutrients than other plant tissues (Parzych, 2022; Álvarez-Alonso et al., 2025) [10,11]. In particular, Zn is highly mobile within plant tissues and tends to accumulate during leaf senescence, which explains the elevated Zn concentrations observed in composts derived from species such as *Juglans regia* and *Betula pendula* (Prescott et al., 2000) [20]. Likewise, the increased Cu concentrations in V2 are likely associated with the formation of stable organo-metallic complexes during composting. Copper readily binds to humic substances produced during humification, reducing its mobility while increasing its retention in compost derived from lignocellulosic feedstocks (Tampio et al., 2016; De Lucia et al., 2013) [29,41].

In contrast to the general increase in Mn concentrations in V2, Larix decidua exhibited lower Mn content. This species-specific response highlights the complex behaviour of Mn during organic matter decomposition. Unlike Zn and Cu, Mn is strongly influenced by oxidation–reduction reactions, microbial transformations, and adsorption onto newly formed humic substances, all of which affect its extractable concentration during compost maturation [1]. Moreover, coniferous litter, including Larix spp., contains relatively high concentrations of lignin and polyphenolic compounds that promote Mn complexation during humification [21]. Consequently, the lower Mn concentration observed in L. decidua compost is more likely attributable to species-specific litter chemistry and Mn stabilisation within organic complexes than to reduced microbial activity or analytical variability.

The observed species-dependent differences in micronutrient accumulation further emphasise the importance of feedstock composition in determining compost quality. Consistent with the findings of Parzych (2022) and Prescott et al. (2000) [10,20], coniferous species generally contributed higher Mn concentrations because of the chemical characteristics of their needles, whereas deciduous species were more effective at enriching compost with Zn and Cu owing to greater variability in foliar nutrient concentrations [10,21]. Conversely, the lower micronutrient concentrations recorded in V1 are most likely explained by a dilution effect resulting from the higher proportion of cattle manure, which typically contains lower concentrations of these trace elements than leaf biomass (Prescott et al., 2000; Tampio et al., 2016) [20,29]. These findings support the concept that compost micronutrient composition is determined by both the chemical characteristics of the feedstock and the biochemical transformations occurring during composting, including mineralisation, humification, metal complexation, and element-specific mobility (Awasthi et al., 2020) [19].

From a practical perspective, the higher micronutrient concentrations in V2 suggest that composts produced predominantly from leaf biomass may serve as valuable organic amendments to improve the micronutrient status of Mn- and Zn-deficient soils. Nevertheless, feedstocks with high Mn or Cu accumulation should be carefully managed to avoid excessive trace-element inputs following repeated applications, particularly in agricultural systems where long-term metal accumulation may become a concern (Álvarez-Alonso et al., 2025) [11]. Overall, these results demonstrate that careful selection of plant species and feedstock composition can be used to optimise the micronutrient value of compost while minimising potential environmental risks.

The higher Pb, Cd, and Ni concentrations observed in the V2 treatment were associated with the greater proportion of pruning biomass incorporated into the compost mixture. Similar trends have been reported in composts containing urban green waste, where plant-derived feedstocks generally contain higher concentrations of trace elements than animal manures (Parzych, 2022; Zhang et al., 2016; De Lucia et al., 2013;) [10,13,41]. Urban tree leaves and young woody tissues may accumulate trace elements through both root uptake from soils and interception of atmospheric particulate matter originating from traffic and other anthropogenic activities (Smith, 2009; Stefanowicz et al., 2016) [15,17]. In addition, deciduous species have been shown to retain greater quantities of airborne particles than coniferous species because of their larger leaf area, higher stomatal density, and thinner cuticles (Sæbø et al., 2012; Zhang et al., 2022) [22,42]. These characteristics may contribute to the elevated concentrations of potentially toxic trace elements frequently reported in urban green waste (Álvarez-Alonso et al., 2025; Parzych, 2022) [10,11].

However, the present study did not determine trace-element concentrations in the original pruning biomass, cattle manure, or surrounding soils, nor did it quantify atmospheric deposition. Consequently, the relative contributions of these potential sources cannot be distinguished, and the observed differences should not be interpreted as direct evidence that atmospheric deposition or pruning biomass alone was responsible for the increased Pb, Cd, and Ni concentrations. Instead, the results indicate that composts prepared with a higher proportion of pruning biomass (V2) contained higher concentrations of these elements than those with a greater proportion of cattle manure (V1), suggesting that feedstock composition played an important role in determining the trace-element content of the final product.

The consistently higher concentrations of Pb, Cd, and Ni in the biomass-rich V2 treatment, despite its substantially lower manure content, support the hypothesis that the composition of the composting materials influenced heavy metal accumulation. This interpretation aligns with previous studies showing that urban pruning residues generally contain higher concentrations of trace elements than livestock manures (Zhang et al., 2016; De Lucia et al., 2013) [13,41]. Nevertheless, definitive identification of trace-element sources requires chemical characterisation of the individual raw materials before composting, together with complementary analyses of soil trace-element status and atmospheric deposition. Such investigations would enable a more robust assessment of the pathways responsible for trace-element accumulation in compost derived from urban green waste.

Although the concentrations of Pb, Cd, and Ni measured in all composts remained below the current regulatory limits, these findings should be interpreted with caution. Heavy metals are not biodegradable and tend to accumulate in soils following repeated compost applications. Consequently, even relatively low metal inputs may contribute to a progressive increase in soil heavy metal concentrations over the long term, particularly in agricultural systems receiving regular organic amendments. Therefore, compliance with regulatory thresholds for individual compost batches does not necessarily eliminate the risk of long-term soil contamination. Sustainable use of composts derived from urban pruning residues should include periodic monitoring of soil heavy metal concentrations and bioavailability, especially under repeated application scenarios, to prevent their gradual accumulation and potential transfer into the food chain [43,44].

The PCA analysis shows that plant residue type is a major determinant of compost chemical variability, yielding two distinct functional patterns for deciduous and coniferous arboricultural biomass. Compost derived from conifer pruning residues exhibits low variability and tightly clustered PCA structures, reflecting the strong influence of refractory compounds—lignin, resins, and phenolics—on decomposition and nutrient availability. In these composts, PCA gradients are driven mainly by differences in metal accumulation and moderate shifts in pH and macronutrient levels, indicating a slow, conservative mineralisation regime that is partially decoupled from nutrient dynamics [21,45,46,47]. In contrast, composts produced from deciduous pruning residues show much broader dispersion along PCA axes, consistent with faster mineralisation driven by higher proportions of labile compounds and basic cations [21,48]. Here, the dominant gradients reveal strong co-variability between macro-elements and metals, suggesting that mobilisation processes are more integrated and strongly controlled by substrate quality [49,50]. Unlike conifers—where metals act mainly as secondary differentiation factors—deciduous systems display tight metal–nutrient coupling, especially under high inputs of plant material, consistent with observations of simultaneous nutrient and metal release in active organic matrices [42]. pH also contributes more strongly to species separation in deciduous composts, indicating a greater influence on element availability [46]. These contrasts show that deciduous pruning residues generate more dynamic but heterogeneous composts, whereas conifer residues produce more stable but nutrient-limited systems. 

## 4. Materials and Methods

**Figure 7:**
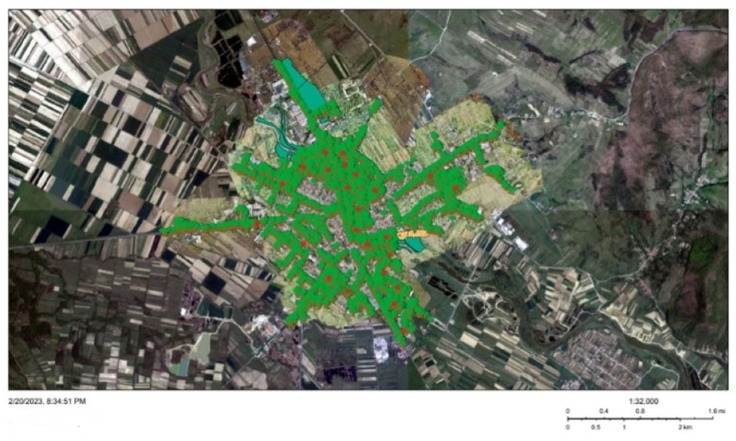
Register of green spaces of the Municipality of Lugoj.

The study was conducted in the municipality of Lugoj, in south-western Romania, on the Timiș River (45°40′28″ N; 21°54′30″ E). According to the municipality’s Green Spaces Register (Figure 7), updated in 2021, the urban dendrological fund comprises 17,765 trees and shrubs.

### 4.1. Experimental Design

For the experiment, 18 tree species commonly found in parks, street alignments and other categories of green spaces managed by the Lugoj City Hall were selected. Of these, 11 were deciduous species: *Catalpa bignonioides* (CB), *Malus purpurea* (MP), *Betula pendula* (BP), *Aesculus hippocastanum* (AH), *Alnus glutinosa* (AG), *Quercus coccinea* (QC), *Platanus × hybrida* (PH), *Acer platanoides* (AP), *Paulownia tomentosa* (PT), *Corylus avellana* (CA), *Juglans regia* (JR), and 7 were coniferous species: *Picea abies* (PA), *Larix decidua* (LD), *Pinus sylvestris* (PS), *Pinus nigra* (PN), *Pinus strobus* (PST), *Pseudotsuga menziesii* (PM), *Taxodium distichum* (TD).

**Figure 8:**
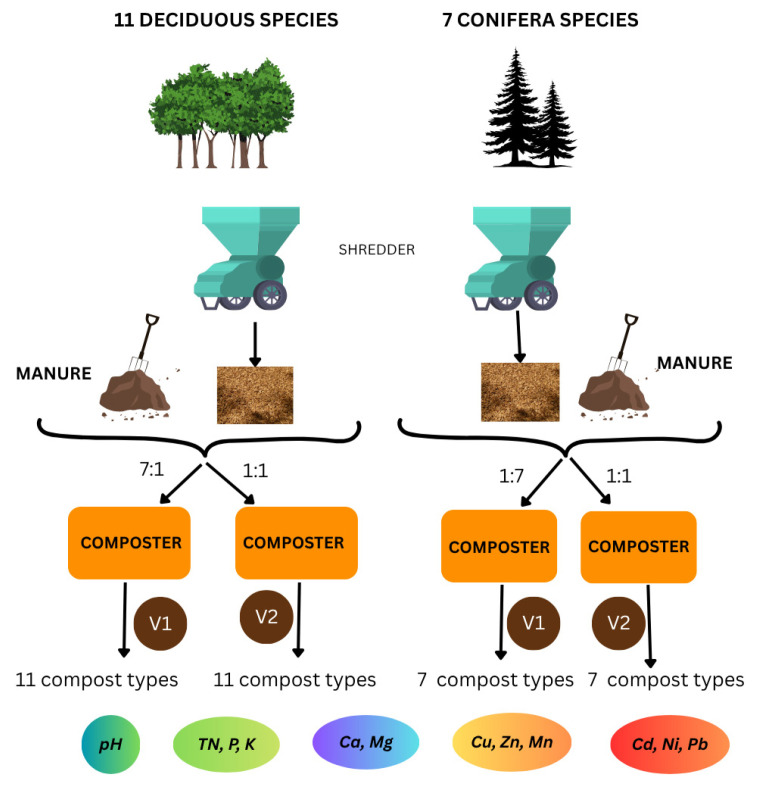
Obtaining compost.

The experiment assessing the composting potential of plant residues generated during the pruning and maintenance of ornamental trees and shrubs was conducted from March to July 2024. A factorial design was employed, comprising two factors (Figure 8). The first factor was the composting ratio, with two levels: V1, a mixture of chopped plant residues and bovine manure at a 1:7 ratio; and V2, a mixture of chopped plant residues and bovine manure at a 1:1 ratio. The second factor was the source of plant residues, comprising 18 ornamental tree species commonly found in the urban green spaces of Lugoj, including 11 deciduous and 7 coniferous species. The combination of the two factors resulted in 36 composting variants, corresponding to all possible combinations of the 18 plant species and the two composting ratios. Vegetation type (deciduous or coniferous) was treated as a botanical grouping variable, with individual species nested within each vegetation category. Each treatment combination was replicated three times, yielding three independent composting units per species–variant combination.

The two composting ratios were selected to represent contrasting proportions of carbon-rich lignocellulosic biomass and nitrogen-rich cattle manure, allowing evaluation of how feedstock composition influences compost quality. Bernal et al. (2009) [1] demonstrated that the proportion of animal manure relative to structural plant residues strongly influences microbial activity, nutrient transformations and compost maturity, emphasizing that the balance between carbon and nitrogen sources is one of the principal determinants of composting efficiency. Similarly, Huang et al. (2008) [32] showed that increasing the proportion of nitrogen-rich material accelerated microbial degradation and promoted higher temperatures during the thermophilic phase, whereas mixtures containing excessive amounts of lignocellulosic material decomposed more slowly because of their high C/N ratio.

Accordingly, the 1:7 plant residue-to-manure ratio (V1) was selected as a manure-dominated treatment to provide abundant nitrogen, moisture and microbial inoculum. Previous studies have shown that cattle manure supplies readily available nutrients and microorganisms that stimulate the biodegradation of cellulose- and lignin-rich plant residues and accelerate the onset of active composting [1,19].

The 1:1 ratio (V2) was selected to represent a practical scenario in which larger quantities of pruning residues generated during urban green-space maintenance are valorised through composting. Awasthi et al. [19] reported that increasing the proportion of lignocellulosic biomass modifies aeration, moisture retention, organic matter degradation and nutrient dynamics, while Pezzolla et al. (2021) [51] highlighted that plant-rich mixtures generally produce composts containing greater amounts of stable humified organic matter, although decomposition may proceed more slowly because of the higher proportion of recalcitrant carbon.

Thus, the selected ratios represent two contrasting composting strategies: a manure-rich formulation expected to favour rapid biodegradation and nutrient mineralisation, and a biomass-rich formulation designed to maximise the recovery of urban pruning residues while evaluating the effect of increased lignocellulosic material on compost characteristics.

### 4.2. Composting and Maturity Assessment

The composting experiments were conducted using pruning residues (branches, leaves, needles, and cones) collected from the 18 tree species under investigation. All plant materials were shredded with a Cippo 25 shredder (Caravaggi, Pontoglio, Italy) to produce particles approximately 1–3 cm in size. The shredded biomass was then manually mixed with fresh bovine manure to prepare the two experimental formulations. 

The homogenised mixtures were placed in a small-scale aerobic composting system comprising perforated PVC containers (2.5 L capacity) positioned above a lower vessel for leachate collection. The perforations (6–8 mm diameter) provided passive aeration, while weekly manual turning further improved oxygen supply and homogenised the material. Leachate generated during the process was collected and discarded; it was not reintroduced into the composting mass.

Composting lasted approximately 14 weeks, from 25 March to 1 July 2024. During the initial phase, the containers were kept in a greenhouse, with ambient temperatures ranging from 16 to 38 °C. As temperatures rose and the risk of overheating became evident, the system was moved to a protected indoor space with controlled temperatures of 11 to 24 °C. The temperature within the composting mass was monitored periodically using a stainless-steel probe thermometer (Step Systems GmbH, Nuremberg, Germany) inserted 10 cm into the material. An active fermentation phase was observed, marked by heat generation and visible structural changes.

Moisture was maintained through periodic water additions, guided by the compost’s physical appearance and environmental conditions. The mixtures were kept within an approximate 50–60% moisture range, with water inputs of 15–30 mL for variant 1 and 100–250 mL for variant 2. To ensure adequate aeration and uniform decomposition, the composting mixtures were manually turned once a week throughout the process.

**Figure 9:**
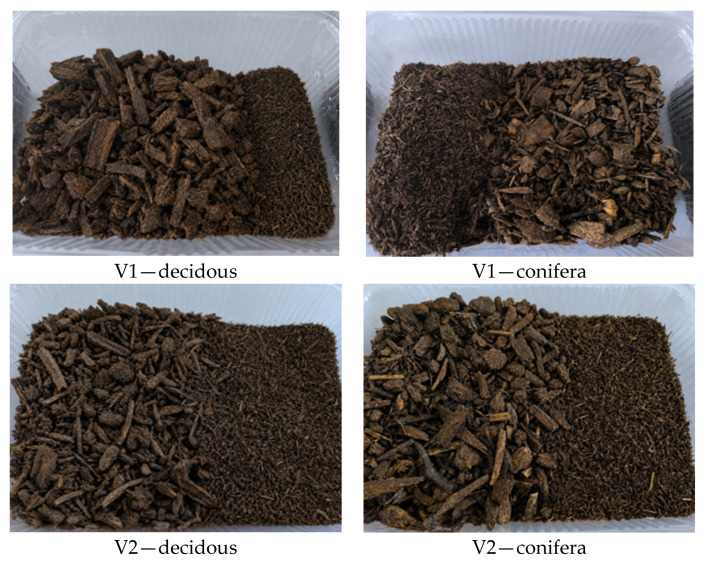
The compost resulted from the application of the two tested recipes.

Compost maturity was assessed throughout the process using physical and organoleptic indicators, with chemical stability criteria providing supporting evidence. As decomposition progressed, the material evolved from a heterogeneous mixture with a vegetal odour to a dark brown–black product with a fine, homogeneous texture and a characteristic earthy smell. In the final stage of the process, no signs of active fermentation or heating were observed, and the original plant structures were no longer distinguishable. At this stage, the composts exhibited a stable pH and a C/N ratio below 20, indicating that they had reached a stable, mature state suitable for subsequent chemical characterization (Figure 9).

After the maturation phase, the composts were transported to the laboratory, air-dried, homogenised, and sieved (2 mm) before analysis. The mature composts were subsequently subjected to chemical analyses, including pH, total nitrogen (TN), total phosphorus (P), total potassium (K), aqua regia-extractable macroelements (Ca and Mg), micronutrients (Mn, Zn and Cu), and pseudo-total potentially toxic trace elements (Ni, Pb and Cd), to evaluate both their agronomic value and environmental safety. Nickel, lead and cadmium were selected because they are among the trace elements most commonly monitored in compost quality assessment and are included in European and national regulations governing compost safety, whereas Mn, Zn and Cu were evaluated separately owing to their essential role as plant micronutrients and their contribution to the agronomic quality of compost.

### 4.3. Chemical Analysis of Compost

The chemical analyses were carried out in the Soil Science Laboratory of the University of Life Sciences “King Mihai I” in Timisoara. 

pH determination. Compost pH was determined using a 1:2.5 soil-to-water ratio. Measurements were performed with a Mettler Toledo digital pH meter (Mettler Toledo, Giessen, Germany) equipped with a combined glass electrode, which was previously calibrated with buffer solutions at pH 4, 7, and 10 (Merck KGaA, Darmstadt, Germany, analytical grade) [52].

Total nitrogen (TN) was determined by the Kjeldahl method in accordance with ISO 11261:1995 [53] using the VELP Kjeldahl System (Velp Scientifica, Usmate, Italy).

Determination of total potassium (K) and total metal content—aqua regia extractable, Ca and Mg, microelements (Mn, Zn, Cu) and pseudo-total heavy metals (Ni, Pb, Cd) [54].

Total metal content was determined by atomic absorption spectrometry (AAS) using a VARIAN 240FS spectrophotometer (Palo Alto, CA, USA). 0.5 g of the dried samples were mineralised with a 3:1 HCl:HNO_3_ mixture in a TOPWAVE Analytic Jena, (Jena, Germany) microwave digester for 60 min at 360 °C to bring the metal ions into solution. After cooling and filtration, the samples were diluted to 50 mL with deionised water. Merck-grade acids were used. For calibration, standard solutions with concentrations ranging from 0.3 to 3 μg·L^−1^ were prepared from a multielement ICP standard solution (1000 mg·L^−1^, Merck Merck KGaA, Darmstadt, Germany). Working conditions were: air:acetylene ratio 13.50:2; nebuliser uptake rate: 5 L·min^−1^; K (404 nm, 4 mA, 0.8 nm); Ca (422.7 nm, 3 mA, 1.2 nm); Mg (282.5 nm, 1.5 mA, 1.2 nm); Mn (279.5 nm, 4 mA, 0.5 nm); Zn (213 nm, 5 mA, 1 nm); Cu (324.8 nm, 4 mA, 0.5 nm); Ni (232 nm, 4 mA, 0.2 nm); Pb (283.3 nm, 10 mA, 1.2 nm); Cd (357.9 nm, 7 mA, 0.2 nm).

Determination of total phosphorus (P) content. Total phosphorus was quantified from the 50 mL aqua regia digest by UV–Vis spectrophotometry on a Cintra spectrophotometer (GBC, Edinburgh, UK). An aliquot of the digest was reacted with an acidic solution of ammonium molybdate and stannous chloride to form ammonium phosphomolybdate, which was then measured calorimetrically at 715 nm. Calibration was performed using standard solutions in the range 2–60 μg·100 mL^−1^, prepared from a 1000 mg·L^−1^ multielement ICP stock solution. All reagents were analytical grade (Merck) [55]. 

### 4.4. Statistical Analysis

Statistical analyses were conducted in Microsoft Excel and OriginPro 2025b, consistent with the experiment’s factorial design. For each chemical parameter, the effects of the composting variant (two levels) and plant species (18 levels), as well as their interaction, were evaluated using two-way ANOVA. When significant main or interaction effects were detected, pairwise comparisons were performed using Tukey’s post hoc test at *p* < 0.05. To complement the species-level analysis, the 18 species were grouped into two botanical categories—deciduous and coniferous—and this classification served as a higher-level grouping variable to compare general trends between vegetation types. Because individual species were nested within vegetation type, these comparisons were interpreted as group-level rather than species-level effects. Principal component analysis (PCA) was applied to obtain an integrated multivariate representation of the compost chemical properties and to explore potential clustering patterns associated with composting variant, plant species, and vegetation type.

## 5. Conclusions

This study demonstrated that both tree species and the proportion of pruning biomass to cattle manure significantly influenced the physicochemical properties and trace-element composition of composts. Composts produced with a higher proportion of pruning biomass (V2; 1:1) contained greater concentrations of micronutrients (Mn, Zn, and Cu) but also higher levels of Pb, Cd, and Ni than those produced with a higher manure content (V1; 1:7). Deciduous species generally yielded composts with higher nutrient enrichment, whereas coniferous species produced more acidic composts with lower trace-element concentrations. Although all composts complied with current regulatory limits for heavy metals, the persistence of trace elements such as Pb, Cd, and Ni warrants careful monitoring of repeated soil applications to prevent their long-term accumulation.

Among the evaluated species, Betula pendula and Platanus × hybrida produced composts with favourable nutrient characteristics, whereas Paulownia tomentosa accumulated comparatively higher concentrations of trace elements and should therefore be used with caution. Overall, the results indicate that appropriate selection of raw materials and the composting ratio is essential for producing composts with desirable agronomic properties while maintaining environmental safety. Further research should evaluate the long-term bioavailability of trace elements and the effects of repeated application of composts derived from urban pruning residues on soil–plant systems.

**Figure 10:**
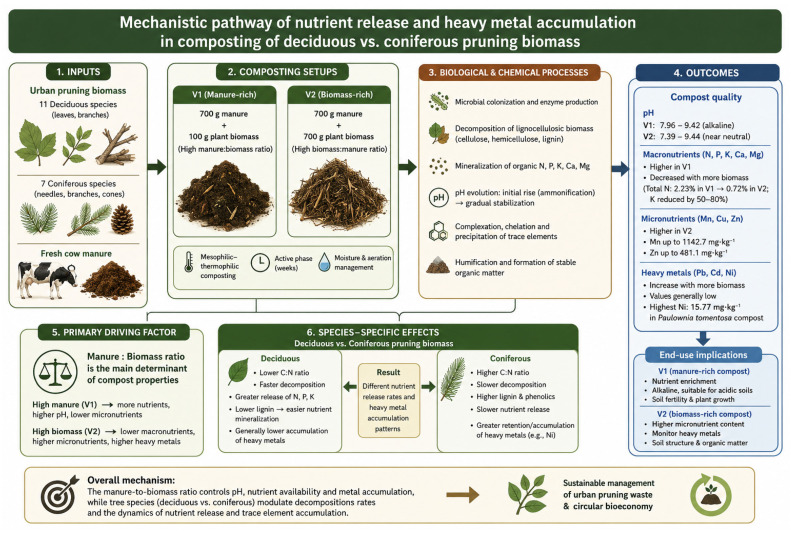
Mechanistic pathway governing nutrient release and trace-element accumulation during the co-composting of deciduous and coniferous urban pruning biomass with cattle manure.

In order to integrate the main experimental findings and clarify the processes governing the chemical composition of the resulting composts we provided a Mechanism diagram (Figure 10) illustrating contrasting raw materials formulations, including the manure-rich V1 treatment (plant biomass ratio of 1:7) and the biomass-rich V2 treatment (1:1), as well as the main biological and chemical processes occurring during composting, such as microbial decomposition, nutrient mineralisation, pH evolution, humification, and trace-element complexation. The manure-to-biomass ratio is the primary determinant of final compost quality: V1 generally produces alkaline, macronutrient-rich composts, whereas V2 favours higher concentrations of Mn, Zn, and Cu but also greater accumulation of Pb, Cd, and Ni. Species-specific plant residues characteristics further modulate these pathways, with deciduous biomass generally decomposing and releasing nutrients more rapidly, whereas coniferous biomass exhibits slower mineralisation due to its higher lignin and phenolic content. These interactions determine the agronomic value, environmental safety, and potential end use of the resulting composts.

## Figures and Tables

**Table 1:** Calcium and magnesium content of the compost.

Species	Ca (mg kg^−1^)		Mg (mg kg^−1^)	
	V1	V2	V1	V2
CONIFERS				
*Larix decidua* (LD)	5001 ± 105 ^b1^	5360 ± 81 ^B1^	2084 ± 86 ^c1^	469 ± 2.3 ^A2^
*Picea abies* (PA)	20,415 ± 783 ^a1^	6521 ± 140 ^B2^	4728 ± 97 ^b1^	471 ± 10.7 ^A2^
*Pinus nigra* (PN)	5868 ± 134 ^cd1^	5974 ± 162 ^D1^	2066 ± 90 ^c1^	485 ± 8.6 ^A2^
*Pinus strobus* (PST)	9715 ± 208 ^c1^	6463 ± 54 ^C2^	5329 ± 150 ^a1^	482 ± 8.4 ^A2^
*Pinus sylvestris* (PSY)	2800 ± 132 ^cd1^	5757 ± 124 ^C2^	1300 ± 68 ^d1^	467 ± 7.4 ^A2^
*Pseudotsuga menziesii* (PM)	5491 ± 130 ^e1^	6019 ± 121 ^C1^	2044 ± 171 ^c1^	477 ± 13.1 ^A2^
*Taxodium distichum* (TD)	4873 ± 48 ^d1^	7105 ± 123 ^A2^	1268 ± 93 ^d1^	478 ± 12.0 ^A2^
DECIDUOUS				
*Acer platanoides* (AP)	17,342 ± 517 ^a1^	6675 ± 157 ^CDE2^	4117 ± 63 ^a1^	482 ± 11.0 ^ABC2^
*Aesculus hippocastanum* (AH)	6477 ± 414 ^de1^	6854 ± 149 ^CD1^	1046 ± 49 ^g1^	474 ± 4.5 ^ABCD2^
*Alnus glutinosa* (AG)	14,918 ± 386 ^b1^	5032 ± 153 ^F2^	3349 ± 60 ^b1^	447 ± 14.1 ^D2^
*Betula pendula* (BP)	6905 ± 197 ^de1^	6319 ± 182 ^E1^	1986 ± 27 ^de1^	466 ± 7.2 ^BCD2^
*Catalpa bignonioides* (CB)	5712 ± 167 ^ef1^	4828 ± 129 ^FG1^	2154 ± 98 ^cd1^	466 ± 12.2 ^BCD2^
*Corylus avellana* (CA)	4982 ± 20 ^fg1^	4553 ± 125 ^G1^	1458 ± 95 ^f1^	458 ± 13.0 ^CD2^
*Juglans regia* (JR)	4638 ± 226 ^g1^	7634 ± 119 ^A2^	1217 ± 39 ^g1^	499 ± 10.8 ^A2^
*Malus x purpurea* (MP)	7019 ± 182 ^d1^	7080 ± 181 ^BC1^	2065 ± 76 ^de1^	480 ± 10.7 ^ABC2^
*Paulownia tomentosa* (PT)	4540 ± 161 ^g1^	3116 ± 120 ^H1^	1571 ± 41 ^f1^	476 ± 6.5 ^ABCD2^
*Platanus hybrida* (PH)	9325 ± 176 ^c1^	6524 ± 107 ^DE2^	2317 ± 105 ^cd1^	490 ± 7.1 ^AB2^
*Quercus coccinea* (QC)	14,582 ± 784 ^b1^	7425 ± 126 ^AB2^	1943 ± 59 ^e1^	485 ± 15.1 ^ABC2^

Lowercase/uppercase letters are used to denote statistically significant differences between groups within the same variant (V1/V2), whereas numbers indicate statistically significant differences between variants.

**Table 2:** Microelement content of the compost.

Species	Variant	Mn (mg kg^−1^)	Cu (mg kg^−1^)	Zn (mg kg^−1^)
CONIFERS				
*Larix decidua* (LD)	V1	606 ± 18.59 ^a1^	16.94 ± 2.37 ^a1^	49.45 ± 6.23 ^a1^
	V2	283 ± 7.91 ^A2^	26.07 ± 2.17 ^A2^	189.30 ± 12.13 ^A2^
*Picea abies* (PA)	V1	244 ± 6.62 ^d1^	14.05 ± 2.38 ^a1^	89.15 ± 7.79 ^b1^
	V2	308 ± 20.27 ^D1^	21.40 ± 2.51 ^AB2^	207.14 ± 17.62 ^B2^
*Pinus nigra* (PN)	V1	497 ± 16.15 ^b1^	15.41 ± 1.70 ^a1^	125.46 ± 16.05 ^d1^
	V2	591 ± 12.28 ^B1^	24.70 ± 2.41 ^BC2^	141.01 ± 17.41 ^BC2^
*Pinus strobus* (PST)	V1	369 ± 19.66 ^c1^	13.83 ± 1.60 ^a1^	123.18 ± 14.65 ^a1^
	V2	1143 ± 78.62 ^C2^	26.66 ± 1.83 ^BC2^	295.10 ± 18.21 ^CD2^
*Pinus sylvestris* (PSY)	V1	242 ± 26.75 ^d1^	11.90 ± 1.91 ^a1^	53.79 ± 6.52 ^bc1^
	V2	254 ± 34.05 ^D1^	19.52 ± 2.53 ^CD2^	129.73 ± 6.59 ^B2^
*Pseudotsuga menziesii* (PM)	V1	271 ± 20.85 ^d1^	16.50 ± 2.22 ^a1^	76.76 ± 3.39 ^cd1^
	V2	340 ± 16.99 ^D1^	31.35 ± 1.66 ^CD2^	202.77 ± 22.44 ^D2^
*Taxodium distichum* (TD)	V1	248 ± 7.09 ^d1^	11.35 ± 1.86 ^a1^	74.97 ± 5.28 ^bcd1^
	V2	360 ± 11.65 ^D2^	32.94 ± 2.07 ^D2^	172.91 ± 25.88 ^BCD2^
DECIDUOUS				
*Acer platanoides* (AP)	V1	347 ± 16.49 ^b1^	27.90 ± 2.20 ^a1^	84.7 ± 4.51 ^cd^
	V2	691 ± 12.11 ^A2^	45.72 ± 3.60 ^B2^	172.0 ± 21.44 ^D2^
*Aesculus hippocastanum* (AH)	V1	276 ± 15.85 ^de1^	27.00 ± 2.70 ^a1^	70.6 ± 7.58 ^cd^
	V2	305 ± 8.71 ^D1^	31.67 ± 3.47 ^CD1^	155.6 ± 9.45 ^DE2^
*Alnus glutinosa* (AG)	V1	485 ± 18.42 ^a1^	24.50 ± 1.40 ^a1^	133.0 ± 21.00 ^a^
	V2	655 ± 31.26 ^A2^	25.08 ± 2.54 ^DE1^	152.7 ± 12.94 ^DE2^
*Betula pendula* (BP)	V1	263 ± 31.48 ^de1^	26.50 ± 2.10 ^a1^	120.4 ± 9.99 ^ab^
	V2	288 ± 11.89 ^DE1^	25.93 ± 3.08 ^DE1^	332.3 ± 29.11 ^B2^
*Catalpa bignonioides* (CB)	V1	205 ± 11.25 ^f1^	24.03 ± 3.28 ^a1^	90.4 ± 22.45 ^bc^
	V2	459 ± 14.75 ^B2^	31.93 ± 1.68 ^CD2^	87.7 ± 5.53 ^F2^
Corylus avellana (CA)	V1	269 ± 17.24 ^de1^	25.94 ± 1.27 ^a1^	54.7 ± 5.08 ^d^
	V2	253 ± 20.07 ^E1^	21.43 ± 1.90 ^E2^	186.2 ± 9.16 ^CD2^
*Juglans regia* (JR)	V1	332 ± 5.88 ^bc1^	22.68 ± 2.13 ^a1^	66.5 ± 4.77 ^cd^
	V2	297 ± 14.24 ^DE1^	34.49 ± 3.80 ^C2^	481.1 ± 31.49 ^A2^
*Malus x purpurea* (MP)	V1	230 ± 8.89 ^ef1^	27.40 ± 2.22 ^a1^	81.1 ± 8.45 ^cd^
	V2	297 ± 24.91 ^DE1^	90.68 ± 2.08 ^A2^	232.1 ± 12.49 ^CD2^
*Paulownia tomentosa* (PT)	V1	353 ± 19.55 ^b1^	27.76 ± 2.39 ^a1^	70.9 ± 5.14 ^cd^
	V2	389 ± 13.57 ^C2^	43.08 ± 2.13 ^B2^	111.3 ± 10.78 ^EF2^
*Platanus hybrida* (PH)	V1	269 ± 12.96 ^de1^	26.71 ± 2.40 ^a1^	63.5 ± 5.25 ^cd^
	V2	274 ± 10.72 ^DE1^	29.31 ± 2.33 ^CD1^	119.4 ± 8.94 ^EF2^
*Quercus coccinea* (QC)	V1	282 ± 18.18 ^cd1^	21.51 ± 2.19 ^a1^	59.4 ± 8.43 ^cd^
	V2	321 ± 19.43 ^D1^	29.26 ± 0.89 ^CD2^	119.4 ± 6.93 ^EF2^

Lowercase/uppercase letters are used to denote statistically significant differences between groups within the same variant (V1/V2), whereas numbers indicate statistically significant differences between variants.

**Table 3:** Heavy metal content of the compost.

Species	Variant	Pb (mg kg^−1^)	Cd (mg· kg^−1^)	Ni (mg kg^−1^)
CONIFEROUS				
*Larix decidua* (LD)	V1	0.223 ± 0.022 ^b1^	0.202 ± 0.026 ^c1^	1.312 ± 0.189 ^a1^
	V2	0.870 ± 0.121 ^CDE2^	0.461 ± 0.011 ^B1^	3.496 ± 0.330 ^A2^
*Picea abies* (PA)	V1	0.106 ± 0.010 ^b1^	0.087 ± 0.010 ^d1^	0.300 ± 0.071 ^cd1^
	V2	0.318 ± 0.020 ^E2^	0.183 ± 0.055 ^C2^	0.408 ± 0.057 ^DE1^
*Pinus nigra* (PN)	V1	0.251 ± 0.025 ^b1^	0.241 ± 0.038 ^bc1^	1.524 ± 0.208 ^a1^
	V2	1.047 ± 0.155 ^CDE2^	0.683 ± 0.018 ^A2^	3.243 ± 0.573 ^A2^
*Pinus strobus* (PST)	V1	0.115 ± 0.023 ^b1^	0.208 ± 0.018 ^c1^	0.890 ± 0.192 ^b1^
	V2	0.680 ± 0.175 ^CDE1^	0.717 ± 0.110 ^A2^	1.860 ± 0.150 ^B2^
*Pinus sylvestris* (PSY)	V1	0.171 ± 0.015 ^b1^	0.279 ± 0.021 ^ab1^	nd
	V2	0.540 ± 0.101 ^DE1^	0.609 ± 0.033 ^AB2^	nd
*Pseudotsuga menziesii* (PM)	V1	0.990 ± 0.105 ^a1^	0.306 ± 0.021 ^a1^	0.895 ± 0.083 ^b1^
	V2	2.607 ± 0.160 ^A2^	0.729 ± 0.071 ^A2^	1.271 ± 0.092 ^BC2^
*Taxodium distichum* (TD)	V1	0.843 ± 0.145 ^a1^	nd	0.554 ± 0.078 ^bc1^
	V2	1.647 ± 0.155 ^B2^	nd	0.795 ± 0.107 ^CD1^
DECIDUOUS				
*Acer platanoides* (AP)	V1	1.263 ± 0.172 ^a1^	0.075 ± 0.013 ^ab1^	1.055 ± 0.198 ^bcd1^
	V2	3.473 ± 0.427 ^A2^	0.107 ± 0.008 ^D2^	2.788 ± 0.294 ^BC2^
*Aesculus hippocastanum* (AH)	V1	0.873 ± 0.130 ^b1^	0.066 ± 0.008 ^ab1^	0.986 ± 0.257 ^cd1^
	V2	1.953 ± 0.191 ^BC2^	0.197 ± 0.010 ^C2^	2.128 ± 0.333 ^CD2^
*Alnus glutinosa* (AG)	V1	nd	0.330 ± 0.056 ^a1^	0.765 ± 0.128 ^d1^
	V2	nd	0.910 ± 0.060 ^A2^	1.196 ± 0.105 ^A2^
*Betula pendula* (BP)	V1	0.352 ± 0.040 ^d1^	0.112 ± 0.013 ^ab1^	0.209 ± 0.042 ^e1^
	V2	1.070 ± 0.075 ^DE2^	0.213 ± 0.007 ^BC1^	0.307 ± 0.068 ^CD1^
*Catalpa bignonioides* (CB)	V1	0.446 ± 0.040 ^d1^	nd	1.453 ± 0.060 ^bc1^
	V2	1.580 ± 0.302 ^BCD2^	nd	3.657 ± 0.319 ^B2^
*Corylus avellana* (CA)	V1	0.090 ± 0.011 ^e1^	0.025 ± 0.006 ^b1^	0.567 ± 0.135 ^de1^
	V2	0.253 ± 0.075 ^F2^	0.080 ± 0.020 ^D2^	0.718 ± 0.082 ^DE1^
*Juglans regia* (JR)	V1	0.404 ± 0.017 ^d1^	nd	1.009 ± 0.113 ^cd1^
	V2	1.493 ± 0.145 ^BCDE2^	nd	2.763 ± 0.139 ^DE2^
*Malus x purpurea* (MP)	V1	0.766 ± 0.024 ^bc1^	0.112 ± 0.013 ^ab1^	1.578 ± 0.143 ^b1^
	V2	2.033 ± 0.135 ^B2^	0.267 ± 0.013 ^BC2^	3.143 ± 0.122 ^E2^
*Paulownia tomentosa* (PT)	V1	0.813 ± 0.115 ^b1^	0.033 ± 0.008 ^b1^	3.563 ± 0.398 ^a1^
	V2	1.963 ± 0.152 ^BC2^	0.096 ± 0.009 ^D1^	15.770 ± 0.505 ^G2^
*Platanus hybrida* (PH)	V1	0.547 ± 0.029 ^cd1^	nd	1.005 ± 0.106 ^cd1^
	V2	1.473 ± 0.125 ^CDE2^	nd	2.276 ± 0.142 ^F2^
*Quercus coccinea* (QC)	V1	0.413 ± 0.041 ^d1^	nd	1.001 ± 0.107 ^cd1^
	V2	0.973 ± 0.075 ^E1^	nd	1.989 ± 0.025 ^FG2^

Lowercase/uppercase letters are used to denote statistically significant differences between groups within the same variant (V1/V2), whereas numbers indicate statistically significant differences between variants.

## Data Availability

The original contributions presented in the study are included in the article. Further inquiries can be directed to the corresponding authors.
